# Physiopathology and Treatment of Obesity and Overweight: A Proposal for a New Anorectic

**DOI:** 10.1155/2024/9587300

**Published:** 2024-03-26

**Authors:** Bruno Silvestrini, Mauro Silvestrini

**Affiliations:** ^1^The Noopolis Foundation, Rome, Italy; ^2^Department of Experimental and Clinical Medicine, Marche Polytechnic University, Ancona, Italy

## Abstract

The “new epidemic,” as WHO calls obesity, is caused by overeating, which, having exceeded the body's actual needs, accumulates in the form of health-damaging fat deposits. Moving more and eating less is the main remedy, but eating belongs to vital instincts, which are beyond the control of reason. In this sense, eating is different from drinking and breathing because without food it is possible to survive for a few weeks, without water for a few days, without oxygen for a few minutes. The first part of this article provides an overview of obesity and its treatment, focusing on the new anorectic anticipated in the title. The second part focuses on compulsive obesity, typically represented by constitutional obesity and food addiction. The article concludes with a discussion of the pharmacological treatment of compulsive diseases, to which some forms of obesity belong.

## 1. Obesity: Different Approaches to Treatment

Obesity and overweight are defined as abnormal or excessive fat accumulation leading to health risks and reduced life expectancy estimated between 2 and 20 years [1–4]. According to WHO, obesity and overweight affect about 60% of the European population and 40–50% worldwide [1, 5].

### 1.1. Possible Approaches for Effective Body Weight Control

All causes of obesity are important, but one prevails over the others: the imbalance between overeating and low physical activity [6]. The imbalance between overeating and low physical activity is one of the results of scientific and technological progress. Food production has more than doubled, while a farmer's labor, which until 2-3 generations ago required up to 500–1000 cal/hour, has dropped to 200–300. In developed countries, millions of young people, adults, and the elderly keep fit through exercise and sports. An elderly person walking or jogging every day is no longer an unusual sight. Walking involves an energy expenditure of about 300 cal/hour. By giving up transportation and walking one hour a day, in one month a person would consume about 9,000 calories, corresponding to more than 1 kg of body fat. There are also millions of people coping with overweight and obesity by eating less. Body fat is worth an average of 8000 cal/kg, while in adults, energy consumption is on the order of 2000 cal/day. It follows that halving your diet would allow you to reduce almost 4 kg of body fat in one month. It is only necessary to have determination and perseverance [7, 8]. “Eating less” is as effective as “moving more,” but as long as you take in all the foods that are essential for the body to function properly. In this sense, the Mediterranean diet is among the most credited [9].  Table 1 shows the possible advantages/disadvantages of the two most common dietary approaches based on food restriction and fasting [10].

A less demanding approach relies on low-digestible foods that swell with water, generating a sense of satiety associated with reduced caloric intake. Another option is to reduce the fat content of foods, but lipoglycides and lipoproteins play a crucial role in the growth, renewal, and maintenance of the body. In addition, some saturated fats play a structural role, typically demonstrated by inhibition of erythrocyte hemolysis [11, 12]. An excess of these so-called “bad fats” causes atheromatous plaque formation, but at adequate doses, they inhibit protein denaturation [13], potentially involved in conformation diseases and dementia [14]. Incidentally, a somewhat similar approach is provided by orlistat, which prevents fat absorption by acting as a lipase inhibitor, thus reducing caloric intake [15]. A different approach involves selectively reducing sugars to induce the body to burn fat. Caution is needed here, too, because sugars play a structural and functional role in the form of glycoproteins and glycolipids. Reducing sugars in the diet is likely to stimulate fat burning. In addition to being the body's basic fuel, sugars play a structural and functional role in the form of glycoproteins and glycolipids. Therefore, a hypoglycemic diet can be just as risky as a hypolipidemic diet [16].

Over the past decade, there has been an increase in the use of various surgical procedures of varying levels of complexity, with the aim of reducing gastric capacity, creating diversions, or a combination of both. Bariatric surgery is the most effective intervention for weight loss and is associated with a favorable impact on obesity-related complications [17]. Unfortunately, despite the initial benefits, increased weight regain occurs in 21% of patients after initial weight loss [18]. In addition, the procedure may carry some long-term risks and requires careful preoperative psychosocial assessment for compliance, ongoing nutritional counseling, and vitamin and micronutrient supplementation [19].

### 1.2. Conventional Anorectics

Anorectics provide support for other measures against obesity and overweight by promoting appetite reduction. Amphetamines remain, despite their side effects, the main model. Their mechanism of action resembles the fight reaction, which in stressful situations triggers a rush of humoral mediators (catecholamines such as adrenaline, indolamines, serotonin, glucocorticoids, mineralocorticoids, and other hormones) that mobilize the body's physical and mental resources, enabling it to perform at its best [20–22]. Energy consumption increases while hunger decreases: this is the dual mechanism of action of the attack reaction and amphetamine [23]. Unfortunately, the physiological reaction has self-regulation that limits its intensity and risks, whereas drugs do not. The consequences can be devastating. The problem first emerged with the death of cyclist Tom Simpson from cardiovascular collapse during the 1967 Tour de France. Since then, amphetamines have been progressively confined to otherwise incurable conditions, such as attention deficit hyperactivity disorder (ADHD), but anorectics continue to proliferate. In addition to numerous amphetamine derivatives and analogs, they include several active substances [24]. Most weight loss drugs work by reducing appetite or increasing satiety. Some do both. The exception is orlistat, which acts by reducing fat absorption.  Table 2 details the mechanism of action and common side effects of antiobesity drugs approved by the FDA for long-term use [15, 25–29]. In order to reduce the risks related to these treatment options, careful consideration of contraindications to the use of each individual anorectics becomes strategic. In addition, recent studies appear to support promising evidence on the possible use of combination therapy as a strategy to optimize efficacy for weight management while minimizing adverse effects [30].

Other emerging approaches should also be considered for future development of obesity treatment. There is evidence that the level of certain bioactive endocrine factors, such as adipokines, can be altered in several metabolism-related diseases. In particular, the results of different investigations support a possible central role for an upregulation of leptin, chemerin, and resistin associated with a downregulation of adiponectin in obesity. A possible approach directed at modulating the imbalance of these adipokines may prove to be an effective future strategy for the treatment of obesity [31]. In addition, recent studies have supported the possibility of expanding pharmacological treatment options for obesity using new methods. In this regard, adipocyte-specific CAMK2 inhibition has been shown to be useful against metabolic dysfunction [32].

### 1.3. A New Anorectic

The anorectic described below is inspired by the idea that hunger reflects a lack of food centrally, as well as peripherally: hence a “mental food,” consisting of glucose and tryptophan administered sublingually, to act on the central nervous system [33]. Glucose is the main energy substrate in the brain, where it binds to receptors in the reward system of the nucleus accumbens [34]. Tryptophan is the precursor of serotonin, which has the ability to reduce hunger and body weight [35–37]. In particular, it activates the brain homeostatic circuit that matches energy intake with energy expenditure and the hedonic circuit, which is involved in the rewarding and motivational aspects of eating [38]. Sublingual administration results in up to a tenfold increase in blood-brain barrier crossing and central effects. More specifically, the new anorectic consists of a sublingual tablet containing glucose and tryptophan at doses approximately 100 and 10 times lower than their normal dietary intake. This composition is notified as a dietary supplement, which as a class has the ability to “maintain, sustain, or optimize a physiological parameter” [39]. In the present case, this parameter consists of appetite regulation according to normal nutritional requirements. Incidentally, the words in quotes are from a Council of Europe document. The new anorectic is distinguished from current ones by its safety and ease of use [33]. These features nominate it as a possible first-line treatment of obesity and overweight.

## 2. Compulsive Obesity

The fight against obesity relies not only on “moving more and eating less,” the decisive measure promoted globally by WHO [40], but also on the rich and varied list of anorexics. Yet, nearly 50% of the human population is obese or overweight. The reasons are to be found in compulsive behaviors, which are beyond the control of reason.

Constitutional obesity is the main example of this. It is a condition rooted in the genome, in which fat accumulation is associated with distinctive physical traits [41, 42]. Its origin dates back to the Darwinian struggle for survival [43], where fat reserve allows hibernating animals to survive the lack of food in winter. It allows undernourished mothers to feed the creatures growing in their wombs. It is still this reserve that facilitated survival during the famines of the past, the seven lean cows of the Bible.

Developed countries are characterized by the abundance of food rather than its scarcity, yet the accumulation of fat related to the constitution persists.

One particular aspect deserving attention is obesity associated with stress. From a physiological point of view, the stress response should mobilize the necessary resources to increase mental and physical performance, allowing for more intense efforts than normal in the face of a difficulty. Specifically, in an effort to expand energy availability, an increase in glycolysis with an increase in blood glucose is an important part of the stress response. Increased blood glucose levels should normally inhibit hunger. However, there is ample evidence that in some individuals, stress, especially when prolonged, can produce a seemingly paradoxical response with increased hunger. Interindividual variability in the relationship between chronic stress and obesity risk has been related to differences in glucocorticoid action. In particular, individuals may have different sensitivity to glucocorticoids, which may influence food consumption and fat accumulation [44]. There are students who under the stress of the exam eat little and lose weight, but there are also those who eat too much and gain weight [45]. This drive is best described by an obese patient who, after several failed attempts, resorted to bariatric surgery: “The surgery was a success. Compared to a year ago, I weigh 60 kg less! I now play sports, regaining the muscle tone I had lost during the rapid decline. It is true that you can find ways to lose excess fat, but keeping fit is another thing. I had surgery on my stomach, but not on my brain! Resisting temptation will be a lifelong war.” A drive rooted in the brain is inevitable, the obese man who gave up tells us, but it doesn't have to be. Here is the testimony of another constitutional obese: “It was hard”-he writes to the doctor thanking him-“but in six months I managed to lose 15 kg. Thanks to the diet, sports, the bet with myself, but also to your help, which has been invaluable.” This is the spirit with which one should approach compulsive obesity. Never give up.

Food addiction, understood as gratification that compensates for life's bitterness and defeats, is the second compulsive eating behavior [46, 47]. Here is how it is described by an obese patient: “Food is the only good thing that gives me a feeling of well-being. Do you want to take that away from me too? I know fat hurts especially with the years, but I am tired and without it I could not live an ugly and unsatisfying life.” Addiction is defined as the subjection to something that deprives the body of a vital function, exercising it instead. Morphine and morphine-like drugs calm and relieve suffering, cocaine and amphetamines give the vigor needed to deal with emergencies, and nicotine supports cognitive processes and relaxes [48, 49]. These physiological capacities are set aside by a substance of abuse, which exercises them instead of the body. This enslavement manifests itself in the withdrawal syndrome in the form of effects that, without their physiological control, are exacerbated: hyperexcitation in the absence of a calming drug, prostration in the absence of excitatory drugs, and mental obnubilation without the nicotine that sustains mental alertness. Treatment of addiction begins with withdrawal syndrome, caused by the absence of the addictive substance. According to WHO, this is a real disease, with signs and symptoms that are dangerous to oneself and others. The first treatment consists of controlled administration of the addictive substance. Examples are oral methadone and buprenorphine, which mimic the effects of morphine in an attenuated form, facilitating cessation. Their equivalent in the case of obesity is provided by appetizing, low-calorie diets [50, 51]. A different approach is disulfiram, which alters alcohol metabolism with side effects (palpitations, headaches, vomiting, etc.) that generate a sense of revulsion toward alcohol [52]. With food, this phenomenon is occasionally observed in cases of indigestion, but so far has not found medical use. The third approach alleviates unpleasant withdrawal symptoms to facilitate abstinence. For example, alcohol deprivation is characterized by insomnia, which can be treated with drugs with opposite effects, such as trazodone [53]. In essence, food addiction obesity is difficult to treat, but it makes use of a pharmacological armamentarium that should not be underestimated.

## 3. Concluding Reflection

The “new epidemic,” as WHO calls obesity, is a varied medical condition that requires thorough diagnosis and comprehensive medical support. “Move more and eat less” is the sovereign remedy, which in the case of simple obesity and overweight can be addressed by relying on psychotherapy, anorexia, and education. The problem of childhood obesity arises mainly at the level of families who do not know the elementary principles of nutrition or encourage sports activities. Compulsive obesity is different. The experience of obesity teaches how delicate and difficult its treatment is, but it also shows that it must always be attempted, perhaps with the help of drugs that did not exist in Cesare Beccaria's time [54]. In this context, the new anorectic described in this article could carve out a nonmarginal role.

## Figures and Tables

**Table 1:** Main advantages and disadvantages of caloric restriction and fasting dietary approaches for obesity.

Dietary regimen	Advantages	Disadvantages
Caloric restriction	(i) Low risk of macronutrients deficit (ii) Long-term effect (iii) Possibility to graduate restriction	(i) No measure to check compliance (ii) Long time needed for effect (iii) Need for intensive patient engagement

Intermittent fasting	(i) Low risk of macronutrients deficit (ii) Fast effect (iii) No need for laboratory monitoring	(i) Interindividual variability (ii) Lack of measures for compliance control (iii) Risk of dehydration (iv) Sustained effect lacking

**Table 2:** Mechanism of action and main side effects of FDA-approved antiobesity medications for long-term use.

Drug	Mechanism of action	Adverse events
Bupropion-naltrexone	Opioid receptor antagonist	Nausea, constipation, headache, vomiting, dizziness, dry mouth, diarrhea, sleep disorder
	DA and NE reuptake inhibitor	
Liraglutide	GLP-1 analog	Increased heart rate, hypoglycemia, constipation, diarrhea, nausea, vomiting, headache
Orlistat	Gastric and pancreatic lipase inhibitor	Oily rectal leakage, abdominal distress and pain, flatulence with discharge, fecal urgency and incontinence, steatorrhea
Phentermine-topiramate	NE agonist/GABA agonist glutamate antagonist	Elevation in heart rate, mood and sleep disorders, cognitive impairment, metabolic acidosis, paresthesia, dry mouth
Semaglutide	GLP-1 analog	Nausea, vomiting, diarrhea, abdominal pain, constipation, headache
Setmelanotide^*∗*^	MC4R agonist	Injection site reactions, hyperpigmentation, nausea, headache, diarrhea, vomiting, abdominal pain
Tirzepatide	GIP/GLP-1 dual agonist	Nausea, diarrhea, decreased appetite, vomiting, constipation, dyspepsia, abdominal pain

DA, dopamine; GABA, gamma-aminobutyric acid; GIP, gastric inhibitory polypeptide; GLP-1, glucagon-like peptide 1; MC4R: melanocortin-4 receptor; NE, norepinephrine. ^*∗*^Only for people aged 6 and older who have obesity due to inherited conditions: (i) pro-opiomelanocortin deficiency; (ii) proprotein subtilisin-kexin type 1 deficiency; (iii) leptin receptor deficiency.

## Data Availability

No data were used to support this study.
